# Bring it on again: antimicrobial stewardship in transplant infectious diseases: updates and new challenges

**DOI:** 10.1017/ash.2023.517

**Published:** 2024-01-11

**Authors:** Helen Tsai, Rachel Bartash, Daniel Burack, Neeraja Swaminathan, Miranda So

**Affiliations:** 1 Division of Infectious Diseases, Department of Medicine, Montefiore Medical Center, Albert Einstein College of Medicine, Bronx, NY, USA; 2 Division of Infectious Diseases, Department of Internal Medicine, University of Utah, Salt Lake City, UT, USA; 3 Sinai Health-University Health Network Antimicrobial Stewardship Program, University Health Network, Toronto, ON, Canada; 4 Leslie Dan Faculty of Pharmacy, University of Toronto, Toronto, ON, Canada; 5 Division of Infectious Diseases, Faculty of Medicine, Dentistry and Health Sciences, University of Melbourne, Melbourne, Australia

## Abstract

Advancement in solid organ transplantation and hematopoietic stem cell transplant continues to improve the health outcomes of patients and widens the number of eligible patients who can benefit from the medical progress. Preserving the effectiveness of antimicrobials remains crucial, as otherwise transplant surgeries would be unsafe due to surgical site infections, and the risk of sepsis with neutropenia would preclude stem cell transplant. In this review, we provide updates on three previously discussed stewardship challenges: febrile neutropenia, *Clostridioides difficile* infection, and asymptomatic bacteriuria. We also offer insight into four new stewardship challenges: the applicability of the “shorter is better” paradigm shift to antimicrobial duration; antibiotic allergy delabeling and desensitization; colonization with multidrug-resistant gram-negative organisms; and management of cytomegalovirus infections. Specifically, data are accumulating for “shorter is better” and antibiotic allergy delabeling in transplant patients, following successes in the general population. Unique to transplant patients are the impact of multidrug-resistant organism colonization on clinical decision-making of antibiotic prophylaxis in transplant procedure and the need for antiviral stewardship in cytomegalovirus. We highlighted the expansion of antimicrobial stewardship interventions as potential solutions for these challenges, as well as gaps in knowledge and opportunities for further research.

## Introduction

Antimicrobial stewardship is uniquely important for solid organ and hematopoietic stem cell transplant recipients, a population who relies heavily on the sustained effectiveness of antimicrobials.^1^ Recent progress in antimicrobial stewardship best practices in transplant patients has been propelled by culminating evidence in antimicrobial treatment optimization and new therapeutic options. In this update to our previous review on antimicrobial stewardship challenges in transplant patients, we provide new insights into febrile neutropenia, *Clostridioides difficile* infections, and asymptomatic bacteriuria in kidney transplant recipients.^2^ We also address four new stewardship challenges: the “shorter is better” paradigm shift in antimicrobial duration, antibiotic allergy delabeling, colonization with gram-negative multidrug-resistant organisms (GN-MDRO), and cytomegalovirus (CMV) stewardship. See Table 1 for summary.

**Table 1.** Summary of new and updates to antimicrobial stewardship challenges and potential solutions

Infectious syndrome(s) and clinical practice in transplant patients	Antimicrobial stewardship challenge(s)	Potential solution(s) and update from literature
Febrile neutropenia in stem cell transplant patients	• Optimal duration, timing of exposure and selection of empirical antibiotic(s), and de-escalation framework during neutropenia continue to be explored for safety and feasibility • Gut dysbiosis and its association with acute graft-versus-host disease (aGVHD)	• Decision to shorten empirical antibiotics during neutropenia should account for fever status and clinical stability based on a recent study • Emerging data suggest antibiotic regimen and timing of exposure may play a role in subsequent development of aGVHD • A thoughtful approach balancing potential risks and benefits recommended
*C. difficile* infection	• Optimal strategies for recurrent *C. difficile* infection continued to be explored with new treatment alternatives • There are limited supporting data thus far in transplant patients, who have multiple non-modifiable risk factors for recurrent infections	• Various strategies include pulse/taper regimens, expanded prophylaxis • Options include vancomycin, fidaxomicin, bezlotoxumab, fecal microbiota transplant with varying supportive data on efficacy and cost-effectiveness
Asymptomatic bacteriuria in kidney transplant recipients	• Optimal microbiology testing strategy for urinary specimen and prescribing of antibiotics post-kidney transplant continue to be examined • Clinical data support not treating ASB in patients beyond 2 months post-transplant, but no recommendations are available for ASB within 2 months of transplantation	• Treatment of ASB with antibiotics in patients within 2 months post-transplant did not decrease risk of urinary tract infections in a recent study • Further research required to support change in practice, especially in context of ongoing antibiotic exposure and multidrug-resistance organisms
Shorter is Better (for duration of antibiotic)	• Paradigm shift in shortening duration of antibiotic therapy is supported by evidence for specific infectious syndromes in the general population. • There is a paucity of data supporting this practice change in the transplant population	• Supporting data from limited studies on neutropenic fever in hematology-oncology/stem cell transplant population; gram-negative bacteremia and urinary tract infections in kidney transplant recipients, and in liver recipients with source control attainment for recurrent cholangitis • New clinical parameters are emerging to determine optimal duration in transplant patients
Antibiotic allergy delabelling	• Delabelling patients with self-reported allergies to penicillins (or other beta-lactams) have been shown to be feasible and safe in the general population, as well as improved outcomes. • Data supporting this practice change in the transplant population continue to be explored	• Successful implementation allergy skin testing in patients with self-reported penicillin and sulfa allergy, with improved outcomes and cost savings • Delabelling programs should ideally be led by infectious diseases specialists and pharmacists
Gram-negative multidrug-resistant organisms colonization and infection	• Transplant patients are disproportionately affected by surgical site infections caused by various GN-MDRO • Best practices to guide the optimal use of peri-operative antibiotics while accounting for local-level and patient-level GN-MDRO risks remained to be defined	• Pre-transplant colonization with GN-MDRO has significant impact on surgical site infections • Rectal colonization status has limited predictive properties to guide surgical antibiotic prophylaxis; data on clinical benefits are being evaluated
Cytomegalovirus prophylaxis and treatment	• CMV infection and syndrome damage allograft tissue and impact long-term survival • No one-size-fits-all strategy for CMV prophylaxis • Optimal roles of alternatives to ganciclovir/valganciclovir, such as letermovir and maribavir continue to be explored • Adverse events and toxicities are major limitations of CMV pharmacotherapy	• Letermovir established in SCT patients for prophylaxis; support for its use in high-risk kidney transplant recipients recently reported, compared to valganciclovir • Letermovir reasonable alternative for patients unable to tolerate valganciclovir due to neutropenia • Maribavir supported in SOT patients with recurrent/refractory CMV infections

## Updates in febrile neutropenia

The “How-Long” study demonstrated that shortening duration of empirical antimicrobial therapy with close monitoring was safe in patients with high-risk febrile neutropenia (FN), including clinically stable neutropenic stem cell transplant (SCT) recipients.^3^ However, a recent open-label, non-inferiority study comparing a short course (72 hours, irrespective of fever) with an extended course (9–14 days, afebrile for 5 days or neutrophil count recovered to ≥500 cells/µL) of empirical antipseudomonal carbapenem yielded contrasting results.^4^ Although the primary composite outcome met non-inferiority criteria, all-cause mortality was significantly higher in the short course arm (3% [5/144] vs. 1% [1/137], adjusted risk difference 2.6%, 95% CI 1.2–4.1%, p<0.0001). The divergence from “How-Long” may be explained by a higher proportion of SCT recipients (72% vs. 55%) and discontinuation of antibiotics before resolution of fever. This contradiction may augment the perceived stewardship challenges.

Conversely, there is growing recognition of the association between peri-SCT gut dysbiosis from antimicrobial exposure and subsequent acute graft-versus-host disease (aGVHD).^5,6^ Rashidi et al. modeled the association using data from a cohort of 2023 allogeneic SCT patients.^7^ The risk of aGVHD following exposure to carbapenems <2 weeks post-allogeneic SCT was high (hazard ratio [HR] 2.75; 95% CI, 1.77–4.28), as was exposure to beta-lactamase inhibitor-penicillin combinations <1 week after transplant (HR, 6.55; 95%CI, 2.35–18.20), suggesting limiting unnecessary antibiotic exposure may prevent aGVHD. Recent surveys described variation in practice regarding de-escalating or discontinuing antibiotics in febrile neutropenia indicating the need for further research.^8,9^

## Updates in *Clostridioides difficile* infection

Targeted antimicrobial and diagnostic stewardship interventions have been successful in reducing rates of *C. difficile* infections (CDI) in solid organ transplant (SOT) and SCT recipients, but optimal strategies to address recurrent CDI (rCDI) deserve further attention. The estimated incidence of rCDI in SOT and SCT recipients ranges from 6.3%–41%,^10^ and risk factors for recurrence are often non-modifiable, including frequent hospitalizations and antibiotic exposure.

With evidence of its superiority over vancomycin in achieving sustained clinical response, fidaxomicin plays a valuable role for those at increased risk of rCDI.^11^ However, the potential incremental benefits of fidaxomicin for transplant recipients are poorly characterized, and two single-center retrospective studies involving SOT and SCT recipients demonstrated no difference in recurrence rates between fidaxomicin versus conventional vancomycin or metronidazole therapy.^12,13^ Randomized control trials of bezlotoxumab for rCDI included immunocompromised patients as determined by medical history or use of immunosuppressive therapy, but transplant status was not detailed, and <4% of the study population were treated with fidaxomicin.^14,15^ Recent studies by Askar et al.^16^ and Hengel et al.^17^ included strong representation of SOT and SCT recipients and demonstrated success of bezlotoxumab in reducing rCDI compared to standard-of-care antibiotics alone. However, only 3.8% and 30% were treated with fidaxomicin, respectively, and the benefit of bezlotoxumab as a co-intervention may be diminished when fidaxomicin is used as initial treatment.^18^

Fidaxomicin, tapered/pulse regimens, prophylactic vancomycin, and bezlotoxumab are appealing therapies for rCDI in transplant patients, but guidance for the best approach is lacking. Identifying clinical factors or biomarkers that predict the success of one CDI treatment over another can potentially guide stewardship practices. Other considerations are cost-effectiveness analyses of combination or sequential treatments and navigating logistical implementation barriers of bezlotoxumab. Fecal microbiota transplant (FMT) is a promising modality to reduce rCDI, and its efficacy and safety in transplant recipients have been illustrated in case series.^19^ Rebyota®, the first FDA-approved fecal microbiota product, is another encouraging therapeutic advancement, though data specific to transplant recipients are minimal.^20^ As data emerge to clarify candidate selection criteria and long-term outcomes, the relationship between fecal microbiota therapies and antimicrobial stewardship is an evolving area of interest.

## Updates in asymptomatic bacteriuria

A stewardship challenge previously highlighted was the uncertainty of management of asymptomatic bacteriuria (ASB) in the first 2 months post-kidney transplantation.^2^ Guidelines do not account for this specific time frame,^21^ which was excluded from previous studies.^22,23^ However, a recent RCT of 80 kidney transplant (KT) recipients with indwelling ureteral catheters found that receiving antibiotics for ASB in the first 2 months post-transplant did not decrease the risk of urinary tract infection (UTI) or pyelonephritis.^24^ Rather, the incidence of UTI (25% vs 10%, *p* = .07) and pyelonephritis (15% vs 2.5%, *p* = .04) were higher among those receiving antibiotics. Approximately 60% of the urinary isolates were *Escherichia coli,* with over half classified as extended-spectrum beta-lactamase-producing (ESBL), emphasizing the importance of judicious antibiotic exposure in this population. Despite its limitations, including small sample size, young age of participants, and underrepresentation of patients with diabetes, this study provided the first data supporting the safety of no treatment for ASB early post-transplant despite presence of an indwelling ureteral catheter. Though larger studies with more diverse patients are needed to enhance the generalizability of these findings, these data should be considered when developing treatment protocols.

## New challenge 1: Is shorter (antimicrobial duration) better?

While recent studies support shorter treatment courses for various clinical syndromes,^25–29^ the applicability of this new paradigm to immunocompromised hosts remains controversial.^30^ While shortening unnecessary duration of antibiotic therapy should be considered in optimizing care, limited efficacy and safety data supporting this practice remain a challenge for transplant providers.

Beyond FN as discussed, data supporting shorter durations of antimicrobial therapy in immunocompromised hosts are limited. Growing evidence suggests that shorter treatment durations are sufficient for uncomplicated gram-negative bacteremia including *Pseudomonas aeruginosa*, but immunocompromised patients only comprised 10%–24% of the studies’ populations, and subgroup analyses have not been reported.^27,28,31,32^ Imlay and Spellberg recently published additional details based on communication with Yahav et al.,^27^ reporting that of 40 KT recipients, there was no difference in a composite outcome among those receiving 7 vs. 14 days of therapy (62% vs 68%).^30^ Data for shorter antibiotic courses for gram-negative bacteremia in neutropenic patients with hematological malignancy or SCT are more variable. A retrospective cohort study of 206 neutropenic patients with hematologic malignancy or SCT with documented gram-negative bacteremia including *Pseudomonas* found no difference in a composite outcome among those receiving shorter duration (<10 days of therapy) of antibiotics compared to longer durations (either 11–14 days or >15 days).^33^In contrast, a retrospective study of 55 allogeneic SCT recipients with *Pseudomonas* infections found a significantly higher rate of recurrence in those who received <14 days of therapy and even <21 days of therapy compared to longer durations.^34^

Shorter durations of therapy may also be appropriate for UTIs in KT recipients and in recurrent cholangitis in liver transplant (LT) recipients. One small retrospective study found no difference in mortality or rates of readmission for complicated UTIs with shorter courses of therapy.^35^ Similarly, a retrospective study evaluated shorter (5 days) versus longer (8 days) antimicrobial therapy in LT recipients with recurrent cholangitis found no difference in the rate of recurrence at 28 days (13.9% vs 19.2%, *p* > 0.2).^36^ Of note, all patients underwent endoscopic retrograde cholangiopancreatography for source control and those with severe infection or sepsis were excluded, limiting the generalizability of its findings. Minimizing the potential negative consequences associated with prolonged antibiotic use is crucial in immunocompromised patients and shorter durations should considered as a stewardship intervention.^30^

## New challenge 2: Antibiotic allergy

The negative impact of self-reported ß-lactam allergy (BLA) described in the general population, including increased rates of multidrug-resistant organisms (eg, MRSA), *C. difficile* infections, longer hospital stays, and higher healthcare costs, has been also been described in transplant patients, albeit less well delineated.^37–42^ Data from two retrospective studies, one consisting of 2,153 transplant patients (SOT or SCT), and another of 1,700 SOT recipients, estimated the prevalence of BLA to be 16%.^37,38^ BLA was associated with greater use of non-beta-lactam alternatives; however, only the study with a combined cohort of SOT and SCT patients demonstrated a trend toward increased mortality in the BLA group.^37^ In a retrospective analysis of 15,489 KT recipients, patients with BLA had significantly higher mean costs of hospitalization and rates of antibiotic-related adverse events compared to those without BLA.^39^ As transplant patients rely heavily on ß-lactam antibiotics for common indications such as SOT surgical prophylaxis, empirical treatment of FN, and chronic GVHD prophylaxis, BLA presents a major barrier to optimized antimicrobials.

Solutions that are effective at addressing the challenge of BLA in the general population may be useful for transplant patients. PEN-FAST is a clinical decision rule based on patient history that stratifies low-risk phenotypes amenable to point-of-care delabeling.^43^ In a study population that included transplant recipients, PEN-FAST identified patients eligible for direct oral challenge, which was non-inferior to the standard two-step skin testing followed by oral challenge.^44^ Penicillin allergy skin testing (PAST) in the transplant population is a valuable, cost-effective tool for antimicrobial stewardship. True rates of penicillin allergy are low.^45^ Even in pre-lung transplant candidates with low lung volumes, PAST was well tolerated with no reported adverse events.^46^ Studies of PAST demonstrated that up to 95% were successfully delabeled from their BLA, and subsequently, penicillins were safely administered in 51% of patients.^47,48^

Sulfa allergy, reported in 5%–11% of immunocompromised patients, has important implications as sulfonamides are commonly prescribed for prophylaxis against opportunistic infections including *Pneumocystis jiroveci* and toxoplasmosis.^40,45^ One study described a protocol-driven approach for SOT patients and found that among 52 patients with reported non-anaphylactic reaction to sulfa medications, 92% successfully completed a desensitization protocol.^49^ Among them, 80% continued to tolerate sulfamethoxazole-trimethoprim >3 months later without adverse events, resulting in an estimated cost savings of $575 per desensitized patient. Gorsline et al. found that sulfa antibiotic delabeling of 11 SOT recipients resulted in an estimated $254–$2910 saved per patient.^50^

Clinicians may administer validated questionnaires such as PEN-FAST to identify patients who can be delabeled outright and those who require allergy testing or desensitization.^43,44^ Desensitization should be conducted pre-transplant, given the often fluctuating clinical status post-transplantation.^46^ Though highly effective with important downstream clinical and stewardship impacts, widespread implementation of PAST and sulfa desensitization is constrained by the paucity of available inpatient allergy immunology specialists.^51^ The training of multidisciplinary antibiotic stewardship teams to perform beta-lactam allergy skin testing is a strategy that has been successful in increasing preferred beta-lactam use without increasing adverse events.^52^ Experts in antibiotic allergy assessment have advocated for a systematic framework to approach antibiotic allergies as standard-of-care pre-transplant, though implementation can be resource-intensive.^53^

## New challenge 3: Gram-Negative Multidrug-Resistant Organisms (GN-MDRO) colonization status and its impact on peri-operative antibiotics

Post-transplant surgical site infections (SSIs) are a significant early post-transplant complication, occurring between 3% and 53% of recipients, depending on the type of organ transplant.^54^ Peri-operative antibiotic prophylaxis (PAP) can prevent transplant-related SSIs, which are associated with prolonged hospitalization, increased morbidity, readmission rate, and graft failure.^55^ However, with the exception of two older RCTs,^56,57^ evidence evaluating the best approach to PAP in organ transplantation is limited to retrospective studies, with conflicting results.^55,58^ Recommendations for antibiotic selection are based on expert opinion, with suggestion to tailor for organ transplant type, individual risk factors, and local epidemiological patterns, rather than high-quality data.^54^ As rates of GN-MDRO rise, particularly in liver transplant recipients,^59^ there is an opportunity to apply antimicrobial stewardship principles to peri-operative antibiotic management in transplant surgery.

Several studies of liver recipients suggest that GN-MDRO rectal colonization is an independent risk factor for post-transplant infections involving these organisms, with one study finding that carriers of extended-spectrum beta-lactamase-producing Enterobacterales (ESBL-E) were 18 times more likely to develop an ESBL-E infection.^60–62^ A potential mitigating strategy is pre-transplant screening to detect candidates with rectal carriage of GN-MDRO to tailor PAP. Freire et al. demonstrated that adjusted prophylaxis was a significant protective factor against GN-MDRO SSI^63^ and Logre et al. found that patients who received intra-operative prophylaxis active against colonizing ESBL-E isolates had a significantly lower rate of post-operative ESBL-related infections (29.8% vs. 63.6%, *p* = 0.04).^64^ However, ESBL-E rectal carriage had a positive predictive value of only 39% for post-transplant ESBL-E infections, and the data supporting the efficacy of targeted PAP in colonized candidates in decreasing ESBL-E-related SSI are limited. This research gap was reflected in the recent ESCMID/EUCIC guidelines, which conditionally recommended the screening for extended-spectrum cephalosporin-resistant Enterobacterales (ESCR-E), carbapenem-resistant *Acinetobacter baumannii* (CRAB), carbapenem-resistant Enterobacterales (CRE), and targeted PAP for liver transplant candidates colonized with ESCR-E based on low level of evidence.^60^ For other SOT, despite a paucity of data, expert opinion suggests candidate screening for GN-MDRO to facilitate infection control practices and consideration of targeted PAP for known ESBL-E colonization.^60^

Screening for GN-MDRO rectal carriage is an important first step, but indiscriminate adoption of this strategy could lead to carbapenem overuse. The timing of pre-operative screening most predictive of post-transplant SSI is not defined, and peri-operative screening culture results may not be available in time to guide PAP.^65^ Rather than depending solely on colonization status, a clinical prediction tool informed by additional risk factors for GN-MDRO SSI offers a more nuanced strategy.^62,64^ For instance, resistant *K. pneumoniae* carriage, long-term quinolone use for spontaneous bacterial peritonitis prophylaxis, antimicrobial treatment for >3 days within the month before liver transplant, and MELD ≥25 are risk factors for post-liver transplant ESBL-E infections in known carriers.^64^ Robust preventative strategies including judicious antimicrobial use pre-transplant and infection control practices are important. Clearly defined outcome metrics such as number needed to treat^65^ and a detailed understanding of the balancing measures associated with targeted PAP, including impact on emerging antibiotic resistance and *C. difficile* infection rates, will be key to well-designed antimicrobial stewardship interventions.

## New challenge 4: Cytomegalovirus (CMV) prophylaxis and treatment

Intravenous ganciclovir (GCV) and its prodrug PO valganciclovir (VGN) are the mainstay for prophylaxis and treatment in SOT and allogeneic SCT recipients. Efficacy of letermovir (LTV) for prophylaxis has been established in allogeneic SCT^66^ and high-risk kidney recipients based on randomized trials.^67^ Prophylaxis with LTV was associated with lower rates of leukopenia, neutropenia, and discontinuation due to adverse events. However, data on LTV as treatment are scarce. Maribavir (MBV) was effective against refractory/resistant (R/R) CMV infections in an open-label study with investigator-assigned treatment in SOT and allogeneic SCT recipients.^68–72^ Data supporting foscarnet (FCN) and cidofovir (CDV) in R/R CMV infections are limited.^69,73–75^

Several challenges impact successful prevention and treatment of CMV, highlighting the need for CMV stewardship. First, adverse events and toxicity of available options limit long-term tolerability, adherence, and efficacy. Acute kidney injury is a common cause for dose-reduction of GCV/VGN, predisposing patients to subtherapeutic levels and breakthrough infection, while neutropenia and thrombocytopenia often result in discontinuation of therapy.^69,74,75^ Although LTV is better tolerated, adverse events such as thrombocytopenia, nausea, and vomiting may still affect long-term adherence.^76,77^ In R/R CMV, nephrotoxicity and myelosuppression curtail prolonged use of FCN and CDV. Second, mutations that confer resistance may emerge in the presence of incomplete viral suppression from subtherapeutic antiviral levels, affecting long-term efficacy.^69^ Associations between prolonged low-level DNAnemia, resistance, and breakthrough infections are being assessed for LTV and MBV.^76,78,79^ Third, clinically relevant pharmacokinetic and pharmacodynamic drug-drug interactions are complex, often involving immunosuppressants, antimicrobials, and chronic medications. Fourth, there remain several knowledge gaps surrounding prophylaxis strategies across clinical scenarios, including optimal duration of universal prophylaxis, frequency of CMV monitoring during and post-prophylaxis, and clinically meaningful viral thresholds to initiate treatment.^69^ Variability in the limits of detection with the new generation of ultrasensitive DNAnemia testing technology makes determining treatment threshold, efficacy target, and diagnosis of R/R CMV challenging.^74^

Jorgenson et al. described a programmatic approach to CMV management in D+/R- abdominal or kidney transplant recipients, which successfully optimized VGN use, minimized GCV resistance through careful monitoring of CMV levels, and improved access to care.^80–82^ Given the high economic and clinical burden of CMV disease, stewardship has the potential to address the above challenges, especially if its success is demonstrated across other transplantations.

## Gaps in knowledge and opportunities for research

While emerging data offer solutions to address those antimicrobial stewardship challenges, high-quality evidence is required for practice change. Optimal antibiotic management for neutropenic fever maximizing protection from breakthrough infections while minimizing adverse events deserves further exploration. Data defining the roles of new high-cost CDI therapy and studies confirming the safety of not treating ASB early post-kidney transplant will be beneficial. Questions regarding how to safely shorten antibiotic courses, efficiently delabel antibiotic allergies, as well as optimal prophylaxis strategies against GN-MDRO can only be answered by high-quality research. Lastly, the generalizability of CMV stewardship programs remains to be evaluated.

## Conclusion

This review highlights current evidence and controversies surrounding seven challenges in transplant infectious diseases. We emphasized the expanded areas where AMS interventions can address the complex needs of transplant patients, as well as crucial opportunities for further research.
